# OP55 Transferability Of Economic Models Within Health Technology Assessment In Central And Eastern Europe: Bridging The Gap

**DOI:** 10.1017/S026646232400117X

**Published:** 2025-01-07

**Authors:** Malwina Hołownia-Voloskova, Grzegorz Obrzut, Jacek Walczak

## Abstract

**Introduction:**

Health technology assessment (HTA) plays a pivotal role in healthcare decision-making, evaluating the cost-effectiveness of emerging technologies. Central and Eastern Europe (CEE) presents a unique context for HTA, marked by diverse healthcare systems, economic variations, and regulatory frameworks. This study addresses the critical issue of transferability of economic models within HTA in CEE, aiming to bridge existing gaps and enhance the region’s capacity for informed decision-making.

**Methods:**

A comprehensive and systematic approach was employed to assess the transferability of economic models in the context of HTA across CEE. We conducted an extensive literature review, analyzed HTA reports, and engaged in expert consultations to understand the nuances of the healthcare systems in the region. Key factors influencing the transferability of economic models, such as healthcare infrastructure, economic disparities, and regulatory landscapes, were systematically evaluated. The study focused on a range of economic models commonly used in HTA, including cost-effectiveness analysis, budget impact analysis, and multiple-criteria decision analysis.

**Results:**

Our findings highlight the intricate dynamics influencing the transferability of economic models within HTA in CEE. While certain economic models exhibit a degree of generalizability across the region, there are notable variations based on specific contextual factors. Economic models designed for Western healthcare systems may not seamlessly translate to the CEE context due to differences in healthcare delivery, patient populations, and policy frameworks. The study identifies critical determinants of transferability, including the level of healthcare infrastructure development, economic disparities among CEE countries, and the diversity in regulatory approaches.

**Conclusions:**

In conclusion, this study emphasizes the need for a nuanced and context-specific approach to the transferability of economic models within HTA in CEE. Bridging the gap in transferability enhances the region’s capacity for evidence-based healthcare resource allocation and contributes to the overall efficiency and sustainability of healthcare systems in CEE.

